# Effects of Increased N Deposition on Leaf Functional Traits of Four Contrasting Tree Species in Northeast China

**DOI:** 10.3390/plants9091231

**Published:** 2020-09-18

**Authors:** Attaullah Khan, Jingjue Sun, Nowsherwan Zarif, Kashif Khan, Muhammad Atif Jamil, Lixue Yang, Brent Clothier, Boris Rewald

**Affiliations:** 1 Key Laboratory of Sustainable Forest Ecosystem Management-Ministry of Education, School of Forestry, Northeast Forestry University, Heilongjiang, Harbin 150040, China; khan.aup252@gmail.com (A.K.); sunjingjue@nefu.edu.cn (J.S.); nowsherwanzarif@nefu.edu.cn (N.Z.); kashifkhanses@nefu.edu.cn (K.K.); atif2017@nefu.edu.cn (M.A.J.); 2 Pakistan Forest Institute Peshawar (PFI), Khyber Pakhtunkhwa, Peshawar 25000, Pakistan; 3 Sustainable Production, New Zealand Institute for Plant & Food Research Limited, Tennent Drive, Palmerston North 4474, New Zealand; Brent.Clothier@plantandfood.co.nz; 4 Forest Ecology, Department for Forest and Soil Sciences, University of Natural Resources and Life Sciences Vienna, Peter-Jordan-Straße 82, 1190 Vienna, Austria; boris.rewald@boku.ac.at

**Keywords:** angiosperms, biomass allocation, gymnosperms, leaf anatomy, leaf morphology, leaf traits, N deposition, tree seedlings, NE China, stomata pore length

## Abstract

Northeast China is persistently affected by heavy nitrogen (N) deposition. Studying the induced variation in leaf traits is pivotal to develop an understanding of the adaptive plasticity of affected species. This study thus assesses effects of increased N deposition on leaf morphological and anatomical traits and their correlation among and with biomass allocation patterns. A factorial experiment was conducted utilizing seedlings of two gymnosperms (*Larix gmelinii*, *Pinus koraiensis*) and two angiosperms (*Fraxinus mandshurica*, *Tilia amurensis*). Leaf mass per area and leaf density decreased and leaf thickness increased under high N deposition but trait interrelations remained stable. In gymnosperms, leaf mass per area was correlated to both leaf thickness and area, while being correlated to leaf density only in angiosperms. Epidermis, mesophyll thickness, conduit and vascular bundle diameter increased. Despite the differences in taxonomic groups and leaf habits, the common patterns of variation suggest that a certain degree of convergence exists between the species’ reaction towards N deposition. However, stomata pore length increased in angiosperms, and decreased in gymnosperms under N deposition. Furthermore, biomass and leaf mass fraction were correlated to leaf traits in gymnosperms only, suggesting a differential coordination of leaf traits and biomass allocation patterns under high N deposition per taxonomic group.

## 1. Introduction

China has been experiencing a high atmospheric nitrogen (N) pollution since the 1980s due to its industrial development [1,2]. Past studies have indicated that central east China is a particular hotspot of both dry and wet N deposition [3,4]. For example, annual wet N deposition over China averaged 13.2 and 21.1 kg ha^−1^ yr^−1^ in the 1980s and 2000s, respectively—indicating an ~60% increase [5,6]. Xu et al. [7], reported average atmospheric NH_3_ concentrations of 6.1 μg N m^−3^, and average NH_4_ and NO_3_ concentrations in precipitation of ~1.6 and 1.3 mg N L^−1^ annually, respectively. In general, NH_3_ was the predominant N species in total dry N deposition (24–72%), compared to 1–43% NO_2_ and 9–37% HNO_3_. Similarly, the annual average wet/bulk deposition fluxes of NH_4_ were 19.3 kg N ha^−1^ yr^−1^—1.3 times greater than NO_3_ deposition, but with a large spatial variation. The total average N deposition in urban areas was greater than in rural areas and averaged 39.9 kg N ha^−1^ yr^−1^ with 23–83% dry deposited [5,7,8]. Although the values of critical N loads in different ecosystems are discussed controversially, it is currently believed that the values of terrestrial ecosystems are approx. 10–20 kg N ha^−1^ yr^−1^ [9]. Jia et al. [5] showed that 61 and 24% of Chinas land cover are affected by N deposits exceeding 10 and 20 kg N ha^−1^ yr^−1^ in the 2000s, respectively; north and north-east China gradually became a center for N deposition, with an average of 25 kg N ha^−1^ yr^−1^ in the 2000s.

Nitrogen is often a growth-limiting nutrient in temperate forests, and, as a result, most tree species have evolved to adapt rapidly to changes in soil N availability. Numerous field observations and nutrient addition experiments have explored the effects of increased N availability on trees; among the common responses to increased N supply are increased photosynthesis and leaf area accompanied by a shift in biomass allocation towards leaves [7,10,11]. However, knowledge regarding the plasticity of morphological and anatomical leaf traits under increased N deposition is still limited. This is surprising as leaf traits are closely related to plant functioning as their variation is affecting key physiological processes.

In particular, leaf mass per area (LMA or its inverse, specific leaf area, SLA), is one of the central variables among the morphological leaf traits. The LMA is highly correlated with leaf processes such as the maximum photosynthetic rate, A_max_ [12,13,14], species’ potential growth rate [15], and ecosystem processes such as decomposition [16,17]. Higher values of LMA (or lower values of SLA) contribute to long leaf life-span, nutrient retention and protection from desiccation, herbivores and frost [18]. In its simplest form, LMA can be broken down into the product of leaf density (LD) and leaf thickness (LT; also expressed as leaf volume to area ratio, or volume (in needles)) [14,19]. As LMA and its related traits are linked to plants’ carbon budgets, they are tightly associated across and within biomes and largely shape the leaf economic spectra [20,21]. While species-specific LMAs were generally found to be positively related to leaf photosynthetic capacity (per leaf area; A_max_/A), LMA and photosynthetic capacity per dry mass (A_max_/DM) correlate weakly in multispecies comparisons [14]. As variability in leaf N contents may partially underlie this variation, it is key to study LMA vs. N relations as depended on the variation of LD and LT [22]. The response of leaf N content to increased N deposition is generally positive but varies largely by species [23]. Earlier, a comprehensive meta-analysis [14], indicated that A_max_/DM was negatively related to LMA because of a negative correlation between A_max_/DM and LD, and that LD was (weakly) negatively correlated to leaf N content.

An important step towards understanding LMA variation is to consider the tissues that shape leaves. Both LT and LD are determined by the composition of different tissues: epidermis, mesophyll and vascular plus sclerenchymatic tissue [24,25,26]. However, while effects of N availability on leaf morphological traits have received considerable attention, studies including accompanying changes of leaf anatomical traits are rare [27]. This is surprising, as changes in, e.g., the size and density of stomata, veins and mesophyll tissue structure affect the performance of photosynthesis and transpiration [28,29,30,31]. As leaves are contributing 30–80% of the whole-plant hydraulic resistance, hydraulic traits have in particular the potential to affect photosynthetic performance—independent of LMA variation [31]. Gymnosperms and angiosperms differ largely in their vein system [32,33], and vessels of angiosperms allow higher leaf hydraulic conductivities than tracheids of gymnosperms—partially underlying higher growth rates and photosynthetic capacities in angiosperms [34,35]. On the other hand, more frequent tissues relate to LMA via shaping LD—the volumetric fraction of the palisade parenchyma was found to be significantly related to LD, and, as an increase in LD may increase the intercellular transfer resistance to CO_2_, affecting A_max_/DM negatively [14]. Earlier studies on the effects of excess N fertilization on leaf anatomical traits revealed that, e.g., *Pinus sylvestris* needles became more mesomorphic, featuring larger needles and a thicker adaxial mesophyll—with potential consequences for the stress tolerance of the species [36]. Interestingly, however, the mesophyll thickness in leaves of *Fraxinus mandshurica* and *Quercus mongolica* saplings was recently reported to be significantly enlarged under moderate N fertilization only but not under a higher N addition rate of 69 kg ha^−1^ yr^−1^ [37]. While this may be indicative of a non-linearity of N addition effects on anatomical leaf traits, it remains open if this is a persistent effect in other woody angiosperms and gymnosperms.

The northeastern part of China is one of the major forest regions in China, comprising highly diverse, temperate forests [38]—subjected to already high N deposition rates. Although natural and plantation forests in the region hold multiple deciduous broadleaved and evergreen tree species, the most commercially important species are *L. gmelinii*, *P. koraiensis* (gymnosperms) and *F. mandshurica* and *T. amurensis* (angiosperms) [39].

This study was conducted in order to obtain insights into the variability of leaf traits of seedlings of these four tree species under further, severely increased N deposition. Our specific aims were to elucidate, if severe N deposition affects the relationships between:

- LMA and its components leaf thickness and density;
- Leaf anatomical and morphological traits, and;
- Leaf traits and plant biomass allocation patterns.

We hypothesize that drastically increased N deposition results (i) in a reduced LMA. However, due to previous, contrasting results in regard to the (main) dependency of LMA on LT and/or LD it remains open if the expected change in LMA will equally depend on changes in both components or not. Further we hypothesize that high N deposition (ii) will increase the thickness of the epidermis and/or the mesophyll—maintaining the correlation of these traits with LMA and its components—while we expect that (iii) hydraulic leaf traits scale independently of LMA changes—resulting in modified trait correlations under control and high N depositions. Last, we hypothesize that drastically increased N deposition results in (iv) drastically altered relations between biomass allocation pattern and leaf morphological and anatomical trait values.

We analyzed these questions at three levels to find general or particular patterns, depending on the (group of) species considered: (a) considering species separately, (b) at the level of taxonomic groups (i.e., within angiosperms and gymnosperms), and (c) combined, representing an overview on four important tree species in north-east China.

## 2. Results

### 2.1. Seedlings’ Biomass and Biomass Distribution per Organ

The total plant biomass of all four species significantly increased under additional N deposition in seedlings at time of harvest (Supplementary Tables S1 and S2), indicating greater relative growth rates under increased N deposition. The root-to-shoot ratio of all four species decreased significantly under additional N deposition, based on a significant decrease in biomass allocation to roots and an increased allocation to leaves; allocation to stems was not affected (Supplementary Table S1). However, plant height and stem collar diameter of all four species significantly increased under additional N deposition (Supplementary Figure S1 and S2), indicating a differentiated biomass distribution within the woody parts of the canopy.

### 2.2. Leaf Morphology

**Figure 1:**
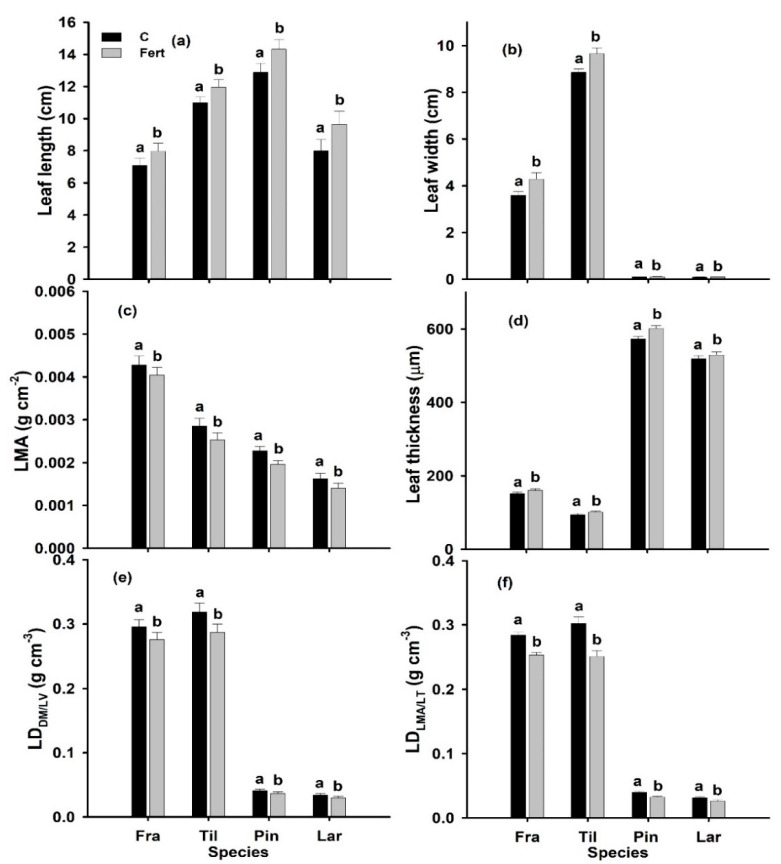
Leaf length (cm) (**a**), leaf width (cm) (**b**), leaf mass per area (LMA: g cm^−2^) (**c**), leaf thickness (µm) (**d**), and leaf density (LD_DM/LV_, g cm^−3^) (**e**), and LD_LMA/LT_ (g cm^−3^) (**f**) of mature sun-exposed leaves/needles of two-year-old seedlings of *Fraxinus mandshurica* (Fra), *Tilia amurensis* (Til), *Pinus koraiensis* (Pin) and *Larix gmelinii* (Lar) at control (C; no additional deposition; black bars) and after 10 g N m^−2^ yr^−1^ (Fert; grey bars) additional nitrogen deposition in NE China. Within species, significant differences between treatments are indicated by different lower-case letters (Tukey’s HSD post hoc; *p* < 0.05; mean ± SE).

Leaf length, leaf width and leaf thickness (LT) of all four species were significantly increased by additional N deposition (Figure 1a,b,d; Table 1). A larger average increase (6.0%) in LT was found in angiosperms (*F. mandshurica*, *T. amurensis*) while the LT of gymnosperms (*P. koraiensis*, *L. gmelinii*) increased on average by 3.5%. In contrast, the leaf mass per area (LMA) and both leaf density indicators (LD_LMA/LT_; LD_DM/LV_) significantly decreased under additional N deposition in all four species (Figure 1c,e,f). The two different estimates of LD were statistically not significantly different (data not shown).

### 2.3. Leaf Anatomy

**Figure 2:**
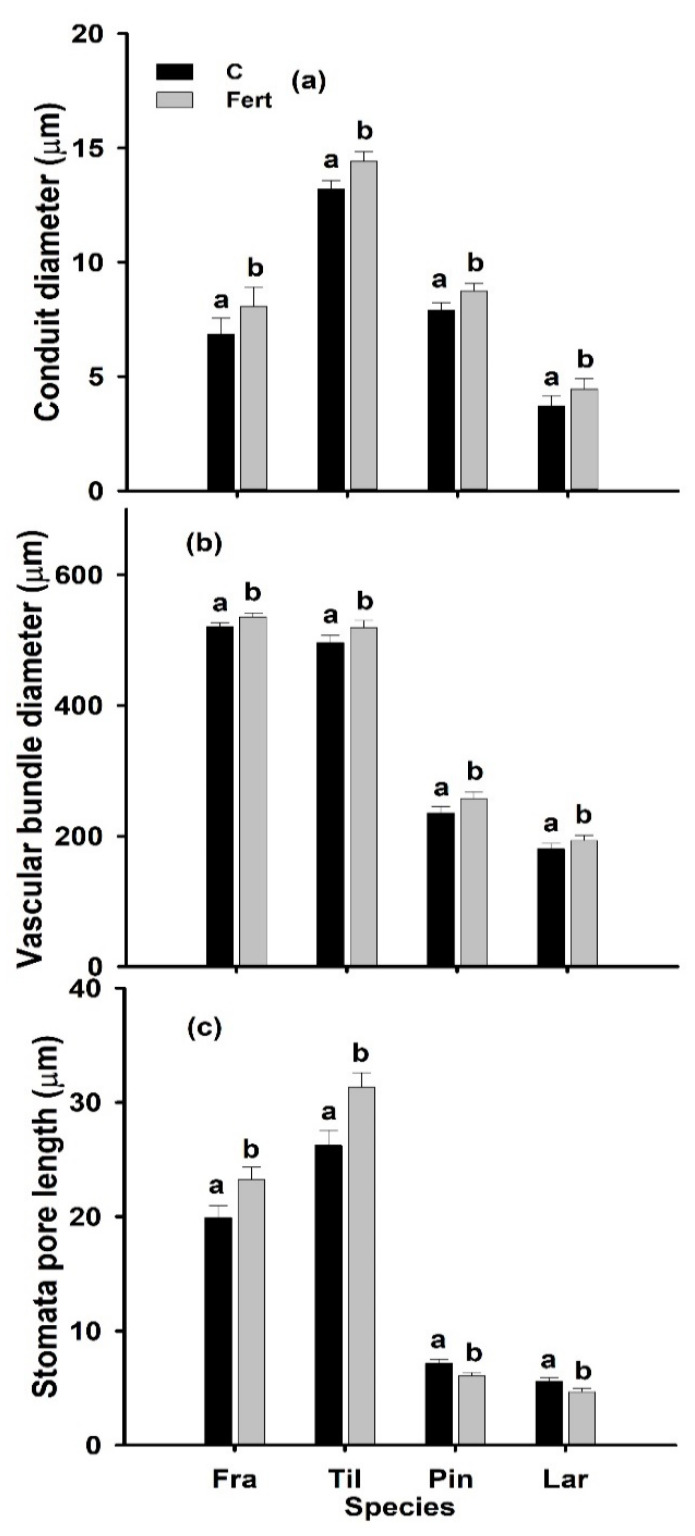
Conduit diameter (µm) (**a**), vascular bundle diameter (µm) (**b**), and stomata pore length (µm) (**c**) of mature, sun-exposed leaves/needles of two-year-old seedlings of *Fraxinus mandshurica* (Fra), *Tilia amurensis* (Til), *Pinus koraiensis* (Pin) and *Larix gmelinii* (Lar) at control (C; no additional N deposition; black bars) and after 10 g-N m^−2^ yr^−1^ (Fert; grey bars) additional nitrogen deposition in NE China. Within species, significant differences between treatments are indicated by different lower-case letters (Tukey’s HSD post hoc; *p* < 0.05; mean ± SE).

Additional N deposition affected most of the studied leaf anatomical traits significantly; significant cross-effects (species × N deposition level) occurred for LD_LMA/LT_ and stomata pore length (Table 1). In angiosperms (*F. mandshurica*, *T. amurensis*), the abaxial and adaxial epidermis, spongy and palisade mesophyll thickness increased significantly under additional N deposition. However, the ratios of palisade to spongy mesophyll thickness, mesophyll thickness to leaf thickness and adaxial to abaxial epidermis remained unaffected by additional N deposition (data not shown). In the gymnosperms (*P. koraiensis*, *L. gmelinii*), the epi- and hypo-dermis thickness and resin duct-diameter increased significantly under additional N deposition (Table 2). In all studied species, the conduit diameter, and vascular-bundle diameter and the stomata pore length of leaves/needles significantly increased under additional N deposition (Figure 2, Table 1).

### 2.4. Correlations of Leaf Functional Traits

Within the leaf traits assessed on all species (Table 3), the leaf width was significantly negatively correlated to the leaf thickness (LT) and positively correlated to the leaf density (LD_DM/LV_), the conduit diameter (CD), the vascular bundle diameter (VBD) and the stomata pore length (SL). Similar, the leaf mass per area (LMA) was significantly negatively correlated to the LT and positively correlated to LD, VBD and SL. The LT was significantly negatively correlated to LD, CD, VBD and SL, while the LD and the VBD were significantly positively correlated to all morphological and anatomical leaf traits besides being negatively correlated to the LT. CD was significantly positively correlated to VBD and SL (Table 3). The correlations within the morphological and anatomical leaf traits were highly stable between the two different N deposition levels.

Looking at the correlations of morphological leaf traits with whole-plant biomass and mass fractions, the leaf length was significantly negatively correlated with plant biomass, root (RMF) and leaf mass fractions (LMF), and the root-to-shoot ratio. It was significantly negatively correlated with the stem mass fraction (SMF; Table 3). Interestingly, LMA was only significantly positively correlated to plant biomass and LMF in the control (C), while no significant correlation was found under additional N deposition. However, the significant negative correlation of the LMA with the SMF persisted under both N regimes. The LT and the LD showed contrasting patterns in regard to their correlation with biomass traits. The LT was significantly negatively correlated to plant biomass and the LMF, while the LD was in both cases positively correlated. However, as LT remained significantly positively correlated to SMF under high N deposition, LD was only negatively correlated to SMF at control. VBD was significantly positively correlated to plant biomass and LMF, and negatively to SMF at control conditions only (Table 3).

Looking at the trait correlations in angiosperms (Supplementary Table S3) and gymnosperms (Supplementary Table S4) separately, the LMA is positively correlated to the LD, and the LT to the LD in gymnosperms only. The LMA, LT and LD were significantly positively correlated to leaf length and width in gymnosperms. In angiosperms, the LT was significantly negatively correlated to leaf length and width, the LMA was significantly negatively correlated to leaf width. Furthermore, the LMA were found to be significantly positively correlated to the CD in gymnosperms and negatively in angiosperms. See Supplementary Tables S3 and S4 for details.

## 3. Discussion

While N is still a growth-limiting nutrient in temperate forests, nutrient imbalance/acidification by excessive N addition—resulting in a lack of phosphorous, magnesium and/or potassium supply—can as well hamper plant growth [39,40]. However, as biomass and height of the four studied species significantly increased under the experimental conditions, without plants showing visible signs for nutrient imbalances, it must be concluded that N was the prime growth-limiting resource for seedlings at the (irrigated) study site. The increase of available N resulted in changes of biomass allocation, a reduced root mass fraction (RMS) and increased leaf mass fraction (LMF), as frequently reported earlier [41,42]—emphasizing the shift of plants’ investment towards light absorbing organs. While some previous studies indicated that greater N deposition rates may reduce the growth and/or decrease the leaf area due to a toxic effect of ammonium on certain tree species, incl. *F. mandshurica* [43,44], this was not the case for the studied species under given experimental conditions—indicating that observed effects on leaf traits are indeed based on a varied N availability.

### 3.1. Effect of N Deposition on LMA and Its Dependency on LT and LD

Given the above, it is not surprising that our results confirm our first hypothesis, namely that severely increased N deposition decreases the LMA among all four tree species. As the LMA captures the tradeoff of a plant’s investment in leaf robustness vs. leaf surface area for photosynthesis [14], the decreased LMA values in our study indicate that the additional N deposition facilitated the latter. While we did not determine leaf N contents, de la Riva and colleagues showed recently that the LMA across woody Mediterranean angiosperms is weakly but significantly negatively correlated to leaf N contents [19]—and thus to N availability [31].

We observed that LMA across species was strongly correlated to both leaf thickness (LT) and leaf density (LD) and that these relations were not affected by N deposition regimes. Previous studies have found the LMA to depend equally on variation LT and LD [14], a stronger correlation between the LT and the leaf volume per area (LVA) [45,46,47], or a stronger dependency of the LMA on the LD [19]. Our results thus rather indicate that variation in LMA across species depend equally on variation in LVA and LD. However, while the N deposition effects on the LT and the LD were surprisingly congruent between the studied gymnosperm and angiosperm species, the latter taxonomic group did not possess a significant correlation between the LMA and the LD (Supplementary Tables S3 and S4). This supports previous findings, namely that the relationships of LMA with LT and LD can hold differences depending on the species (e.g., (evergreen) gymnosperms, deciduous angiosperms)—as indicated earlier [19,46]. This is further supported by the correlation of LT and LD across groups and within gymnosperms but not angiosperms. However, in our study both taxonomic groups maintained more or less similar morphological leaf trait relations—independent of N deposition regimes. This common pattern between different leaf habits suggests that a certain degree of convergence within leaf structures exists in temperate woody species [19,46]. This may be explained by commonly thought consequences of LMA variation; for example, higher LMA (and LD) contribute to higher nutrient retention and protection from desiccation [48] and herbivores [49], while a reduced LMA (and LD) contribute to an efficient resource use and assimilative capacity under optimal conditions [19,50]. However, the recent results of Jin et al. [45,51], serve as a reminder that one must be carefully to deduct long-term functional modifications by increased N deposition from short-term acclimation studies; 16-year of N addition to a *Larix gmelinii* plantation did neither significantly modify LMA nor increased the photosynthetic capacity.

As a technical note, we observed that the LD_DM/LV_ was slightly (but not significantly) greater than the LD_LMA/LT_; the measured values might be influenced by small air-bubbles which formed alongside the leaf cuticle [52]. In addition, the lower LD_LMA/LT_ values may be due to the leaf veins, that are thicker than photosynthetic tissue but not captured by leaf thickness measurements. Consequently, LD_DM/LV_ and LD_LMA/LT_ possessed slightly different correlations with LMA (data not shown) but seem in general both equally suitable to represent LD.

### 3.2. Effects of Additional N Deposition on Leaf Anatomical and Hydraulic Traits and Their Relationship with LT and LD

In partial accordance with previous studies [19,47], our results show that for deciduous species, variation in LT is best explained by variation in palisade and spongy mesophyll thickness and adaxial epidermis thickness. The former could be explained by changes in the number of mesophyll cell layers and/or in cell size [46,53]; the strong relationship between mesophyll thickness and LVA could be related to high rates of A_Max_/DM, which may allow to be more competitive during the (short) growing season [30]. In both gymnosperms and angiosperms, the production of additional mesophyll tissue under additional N deposition may furthermore increase the chlorophyll content of the leaves and mesophyll surface areas—ultimately increasing the photosynthetic capacity—as shown frequently for several woody species including *F. mandshurica* [37,54,55,56,57]. However, the dependency of LT on adaxial epidermis thickness, which is in contrast to previous studies [19], may be related to an adaptation of the deciduous angiosperms to the relatively low precipitation during the growing season—inferring a higher leaf resistance to water diffusion. This could be though to be analog to finding in sclerophyllous evergreen leaves [58], where LMA and LT were also found to be correlated to structural tissues (vascular and sclerenchymatic); in this study, we found a correlation of conduit diameter (and partially vascular bundle diameter) to LT and/or LD. Correlations were more consistent in gymnosperms, likely because evergreens such as *P. koraiensis* often hold more mechanical tissues which could help avoiding damage as result of frost or drought [59]. In previous studies LD of woody species was furthermore found negatively related to the fractions of epidermis and mesophyll but positively related to the fraction of sclerified tissues [19,20,21,22,23,24,25,26,27,28,29,30,31,32,33,34,35,36,37,38,39,40,41,42,43,44,45,46,47]. In partial contrast to our second hypothesis we did not find correlations of LD (and LT) with epidermis and mesophyll thicknesses in the studied gymnosperms; variable cell sizes and number [19,60] and air spaces may thus explain differences in LD (and LT) beside changes in structural tissue fractions [14,46]. Beside the structural aspect, the increase in CD and VBD under increased N deposition is likely directly linked to a higher transport capacity of water and solutes [56,61]—as required in larger leaves with a potentially greater transpiration [62]. Interestingly, stomatal pore length (SL) increased under increased N deposition in angiosperms only, while decreasing in gymnosperms. In accordance, Zhu et al. [37], demonstrated that the SL of *F. mandschurica* and *Q. mongolica* saplings increased significantly after N addition—resulting in greater stomatal opening and transpiration rates. We can only speculate if the decreasing SL of gymnosperms is counter-balanced by increased stomatal densities—making future studies necessary. As hydraulic traits have the potential to affect photosynthetic performance independent of LMA [31], and leaf hydraulic conductance was recently found to be modified by long-tern N fertilization beside stable LMA [45,51]—we hypothesized that hydraulic leaf traits scale independently of LT and LD changes—resulting in modified trait correlations under control and high N depositions. Our results suggest, however, that in the studied system the changes in leaf hydraulic traits, particular VBD, are highly correlated with leaf morphological traits in all four species—contradicting our hypothesis. We speculate that the continuous irrigation of plots, avoiding water stress, has contributed to the tight synchronization of hydraulic and morphological traits—an optimal relation between water conducting elements and transpiring surfaces is likely a function soil moisture.

Finally, the greater resin ducts of gymnosperms under N deposition might indicate an increased capacity for resin secretion and may thus be helpful in increasing the defense against injury and insect/pathogen attacks [63]. In accordance to our results, Jokela et al. [36], reported that N fertilization increased the thickness of the epithelial cells in the resin ducts of *Pinus sylvestris* needles. Increased defense measures against pathogens and herbivores are highly relevant as leaves with greater N contents and lower LMAs are often favored by leaf herbivores [49].

### 3.3. Correlation of LMA with Biomass Allocation

Last, we hypothesize that increased N deposition results in altered relations between plant biomass allocation pattern and leaf morphological and anatomical traits. Indeed, the LMA was found to be correlated to LMF and plant biomass under normal N deposition rates (control) only, but not under additional N deposition. This seems surprising as both the LT and the LD remained correlated with the plant biomass and the LMF under both N deposition levels—although to a lesser extent. However, the taxonomic group’ specific analyses revealed that a significant correlation under either N level persists in gymnosperms only. Duursma and Falster [64] showed that LMA, not total leaf area, drives differences in aboveground biomass distribution among woody plant functional types, and that LMA is more sensitive to environmental changes than other traits shaping the plant biomass allocation above ground. In our study, the decoupled plasticity of LMA in relation to biomass allocation traits is evidenced by the varying correlations with mass fractions under altered N regimes. Our results thus suggest rethinking the way LMA is used in growth models, namely that the correlations between LMA and biomass distribution pattern reported here are highly driven by N availability. In addition, factors such as water availability or competition might alter these relations [65,66]. In the light of the considerable effects of N availability on the plant hydraulic system, it is not surprisingly that the correlations between plant biomass allocation and the hydraulic traits VBD and CD were also partially altered by the N deposition level.

### 3.4. Conclusions

Our results indicate that heavy N deposition significantly increased the LT, and decreased the LMA and the LD across four species differing largely in taxonomy and leaf habit. They confirm further, that LMA variation was strongly correlated to both LD and LT in gymnosperms, and LT in angiosperms, through differences in anatomical composition. While it was no surprise that LT differences in deciduous angiosperms were highly correlated to mesophyll thickness, the correlation with the adaxial epidermis thickness in the studied angiosperms, and the correlation between hydraulic traits and LT and LD in gymnosperms and across species did not match our hypotheses. Despite those differences, the common patterns of trait variation—epidermis, mesophyll thickness, conduit and vascular bundle diameter increased under N deposition in all four species—suggest that a certain degree of convergence exists between the species’ reaction norm. However, leaf traits were significantly related to plant biomass and leaf mass fraction in gymnosperms only, a differential coordination of leaf traits and biomass allocation patterns under high N deposition per taxonomic or leaf habit group seem likely. Our results are thus not fully in accordance with some previous results, stating that the plant biomass above ground is more plastically linked to leaf morphological relative to that of leaf anatomical traits [64]-but emphasize that anatomical and morphological traits of angiosperms vary independent of (leaf) biomass under varied N supply while being highly correlated in gymnosperms.

## 4. Materials and Methods

### 4.1. Research Site

The current research experiment was conducted at the Jiansanjiang Plant Nursery in Heilongjiang, China (47°15′21.0″ N, 132°37′35.0″ E). The nursery is situated in a temperate region with a monsoon climate, an average annual air temperature of 1–2 °C, and a mean summer air temperature of 20–24 °C. The average annual rainfall is 550–600 mm (with monthly maxima in June–August), the annual daylight hours are 2260~2449 h, and the frost-free period is 110–135 d. The soil is classified as chernozemic; basic soil characteristics are a pH (H_2_O) of 6.2, higher proportions of humus, ammonia, phosphoric acids, phosphorus and a greater field capacity.

### 4.2. Experimental Design

*Fraxinus mandshurica* Rupr. (Manchurian ash; Fra), *Tilia amurensis* Rupr. (Amur lime; Til), *Pinus koraiensis* Siebold and Zucc. (Korean pine; Pin) and *Larix gmelinii* (Rupr.) Rupr. (Dahurian larch; Lar) are the major species of temperate forests in Northeast China [67]. These species contrast largely in taxonomy, leaf habit and ecology, as ash and lime are deciduous, broad-leaf angiosperms, while larch and pine are an evergreen gymnosperm.

One-year-old seedlings of uniform sized in terms of plant height were transplanted in May 2018 into plots sized 1 m^2^. Each plot consisted of fifty (50) seedlings and each species was planted into six plots. All the plots were surrounded by an 80 cm deep trench lined with a plastic sheet to prevent entry of water, fertilizers and roots from adjacent plots. One month after planting, two (2) levels of nitrogen (N) deposition were applied to three plots each per species: 0 g N m^−2^ yr^−1^ (Control; C), and 10 g N m^−2^ yr^−1^ (100 kg N ha^−1^ yr^−1^, high, additional N deposition; Fert). Ammonium nitrate (NH_4_NO_3_) was applied—dissolved in irrigation water—as a N source in two split doses on the soil surface—50% in June, and 50% in July 2018 [68]. Two plots for each N level were established per species in a randomized design, and the two-factorial experiment was set with four species and two levels of nitrogen. All 24 plots were carefully irrigated with tap water as needed, preventing leaching.

### 4.3. Seedling Biomass Above and Below Ground

Stem diameter and height of fifteen randomly selected seedlings per plot (excluding trees at the outer boundary/next to the trench) were measured monthly (June, July, August, September) after re-planting in the nursery. For height measurement, the stem was carefully pulled straight from the soil surface up to the tallest apical bud. Stem diameter was measured by taking the orthogonal diameter using digital calipers (MeasumaX IP54, Peterborough, TO, Canada).

For biomass determination, ten randomly selected seedlings per plot (excluding trees at the outer boundary/next to the trench) were harvested during the last week of September 2018, and individually separated into leaf, stem and roots, then dried (60 °C, 72 h), and weighed (±0.0001 g). Root systems were excavated using a hand shovel, with careful sampling all roots to a maximum soil depth of 60 cm. Total biomass, leaf (LMF), stem (SMF) and root mass fractions (RMF), and the root-to-shoot ratio (root:shoot) were calculated based on dry mass per plant [69].

### 4.4. Leaf Morphology

The leaf samples were collected on the same day in late September 2018 from three randomly-selected seedlings located in the middle of each plot (n = 6). For the angiosperms *(F. mandshurica*, *T. amurensis*) five fully developed leaves per seedlings were collected, while for the gymnosperms (*P. koraiensis*, *L. gmelinii*) 50 mature needles were harvested per seedling at the exterior canopy (i.e., ‘sun leaves’) on current year shoots. In the lab, the samples were stored (2 °C) before being imaged with a scanner (gray-scale, 600 dpi; Epson-Expression 10000XL, Epson, Japan). Leaf area (cm^2^), leaf width (cm), and leaf length (cm) were calculated using the software Motic Image Advanced v.3.2 (Motic Corp., Zhejiang, China). Leaf thickness (LT, cm) was measured, using multiple 8-μm-thick cross-sections and the Motic Images Advanced v. 3.2, software (see ‘anatomy section’ below). Leaf volume (LV; cm^3^) was derived by immersing fresh leaves in a beaker with water, placed on a balance—using Archimedes’ principle [52]. Subsequently, all samples were separately oven dried (65 °C, 48 h), and weighed (±0.0001 g) to determine the dry mass (g; DM). Leaf mass per area (LMA; g cm^−2^) was calculated by dividing DM by the leaf area [19]. The leaf density (LD) was calculated in two-ways, either by dividing DM by LV (LD_DM/LV_; g cm^−3^), or by dividing LMA by LT (LD_LMA/LT_) [52,70].

### 4.5. Leaf Tissue Anatomy and Stomata

For anatomical measurements, ten mature ‘sun’ leaves or needles (from the upper canopy) were randomly selected per species and treatment, fixed in Formalin-Aceto-Alcohol (FAA) solution (90 mL 50% ethanol, 5 mL 37% methanol and 5 mL 100% glacial acetic acid), and stored cold (4 °C). In the lab, leaves segments and main vein (only in angiosperms; 3–4 per leaf) and needles, were randomly selected per plot and treatment, stained with fast green (1%), and safranin (2%), and dehydrated in 70, 85, 95 and 100% methanol, successively. Subsequently, segments of leaves/needles of all species and treatments were embedded in paraffin. From each segment, multiple 8-μm-thick cross-sections were made using a rotary microtome (KD-202, KEDEE, China) [71]. The cross-sections were made close to the midrib (in angiosperms) while the middle section of needles was used in gymnosperms; cross-sections were subsequently imaged with a compound microscope (40–1000×; Olympus Corporation, BX-51, Tokyo, Japan). Leaf anatomical traits of the angiosperms were spongy mesophyll thickness (SMT: µm), palisade mesophyll thickness (PMT; µm), and abaxial (ABE) and adaxial (ADE) epidermis thickness (µm). In the gymnosperms, a combined epi-hypodermis thickness (EHT; µm), mesophyll thickness (MT; µm), and resin-duct diameter (RD; µm) were measured. Hydraulic traits measured for all species were conduit diameter (CD; µm), and vascular bundle diameter (VBD; µm). All anatomical traits were determined with the software Motic Images Advanced v. 3.2 (as above).

For leaf stomata analysis, six branches per species were cut, placed in wet filter paper, wrapped in black plastic bags, placed in an icebox, and transported to the laboratory. A total of 10 mature leaves/needles per species and treatment were excised under water and saturated in distilled water overnight. The next day, in angiosperms, the trichomes on the abaxial surfaces were removed with adhesive tape and a layer of nail polish was applied close to the midrib, while in gymnosperms a layer of nail polish was directly applied on the leaf surface. The dry nail polish was collected with adhesive tape, placed on a microscopic slide and imaged using a compound microscope (as above). A total of 150 stomata (on 10 leaves/needles per species and treatment) were analyzed for stomata pore length (SL; µm) with the program Motic Images Advanced v. 3.2, software (as above) [72].

### 4.6. Statistical Analysis

A two-way ANOVA was used on normally distributed data (Shapiro-Wilk test), to investigate the effects of species, N deposition and their interaction on trait values. Homogeneity of variances was tested with Levene’s test. The mean and standard error (SE) for leaf width, length and LMA, LT, LD, and the angiosperms (i.e., PMT, SMT, ADE, ABE) and the gymnosperms’ leaf anatomy (i.e., EHT, MT, and RD), and the leaf hydraulic traits (CD, VBD, and SL) are given. Trait values between N deposition levels per species were compared using a one-way ANOVA followed by a post-hoc test (Tukey’s HSD). Pearson’s correlation coefficients were calculated to determine positive or negative correlations within leaf traits and between leaf traits and biomass parameters—for all species and separately for angio- and gymnosperms—per treatments. All statistical analysis was carried out in the PC program R, v.3.6.1 [73]. Sigma Plots v.12.5 (Systat software Inc., San Jose, CA, USA) was used for creating the figures. If not noted otherwise, a significance level of 0.05 is reported.

## Figures and Tables

**Table 1:** The ANOVA results for tree species, N deposition level, and their interaction effects on leaf morphological and anatomical traits of two-year-old seedlings of *Fraxinus mandshurica*, *Tilia amurensis* (angiosperms), *Pinus koraiensis* and *Larix gmelinii* (gymnosperms) at two levels of nitrogen (N) deposition (i.e., control and 10 g N m^−2^ yr^−1^ additional N deposition) in NE China. LMA, leaf mass per area; LT, leaf thickness; LD_LMA/LT_, leaf density measured; LD_DM/LV_; leaf density estimated, CD, conduit diameter; VBD, vascular bundle diameter; SL, stomata pore length (see text for details).

Source of Variation	df	Leaf Length (cm)	Leaf Width (cm)	LMA (g cm^−2^)	LT (µm)	LD_DM/LV_ (g cm^−3^)	LD_LMA/LT_ (g cm^−3^)	CD (µm)	VBD (µm)	SL (µm)
Species	3	**˂0.001**	**˂0.001**	**˂0.001**	**˂0.001**	**˂0.001**	**˂0.001**	**˂0.001**	**˂0.001**	**˂0.001**
N Deposition	1	**0.008**	**0.007**	**0.021**	**0.009**	**0.030**	**0.001**	**0.015**	**0.012**	**0.020**
Spec × N	3	0.891	0.057	0.980	0.357	0.386	**0.002**	0.950	0.918	**0.005**

*p*-values in bold indicate significant effects.

**Table 2:** Anatomical traits of mature, sun-exposed leaves/needles of two-year-old seedlings of *Fraxinus mandshurica*, *Tilia amurensis* (angiosperms), *Pinus koraiensis* and *Larix gmelinii* (gymnosperms) at control (C; no additional nitrogen (N) deposition) and after 10 g N m^−2^ yr^−1^ (Fert) additional N deposition in NE China.

Species	N Deposition Level	Palisade-	Spongy-	Adaxial	Abaxial	
		Mesophyll Thickness (µm)		Epidermis Thickness (µm)		
*F. mandshurica*	C	81.5 ± 2.4 a	57.4 ± 1.8 a	17.8 ± 0.8 a	7.9 ± 0.5 a	
	Fert	89.3 ± 2.6 b	63.6 ± 1.5 b	18.8 ± 0.7 b	8.5 ± 0.4 b	
*T. amurensis*	C	24.7 ± 1.0 a	46.3 ± 2.1 a	15.7 ± 0.7 a	8.1 ± 0.3 a	
	Fert	28.9 ± 1.2 b	52.6 ± 2.3 b	16.8 ± 0.7 b	8.8 ± 0.3 b	
		**Mesophyll thickness (µm)**		**Epi-hypodermis thickness (µm)**		**Resin duct diameter (µm)**
*P. koraiensis*	C	25.4 ± 1.3 a		12.5 ± 1.1 a		46.7 ± 1.2 a
	Fert	27.8 ± 0.6 b		13.4 ± 1.6 a		49.6 ± 1.2 b
*L. gmelinii*	C	26.9 ± 0.8 a		21.9 ± 1.0 a		25.5 ± 0.9 a
	Fert	30.1 ± 1.3 b		23.8 ± 1.4 b		28.0 ± 0.5 b

Note: significant differences between treatments per species and trait are indicated by different lower-case letters (Tukey’s HSD post hoc; *p* < 0.05; mean ± SE).

**Table 3:** Pearson’s correlation coefficients of selected leaf morphological and anatomical traits, biomass and mass fractions of two-year-old seedlings of *Fraxinus mandshurica*, *Tilia amurensis*, *Larix gmelinii*, and *Pinus koraiensis* at control (C; no additional deposition) and after 10 g N m^−2^ yr^−1^ (Fert) additional nitrogen deposition in NE China. Abbreviations: leaf mass per area (LMA), leaf thickness (LT), leaf density measured (LD_DM/LV_), conduit diameter (CD), vascular bundle diameter (VBD), stomata pore length (SL), root mass fraction (RMF), stem mass fraction (SMF) and leaf mass fraction (LMF), see text for details. See Supplementary Tables S3 and S4 and for separate Pearson correlation tables for angiosperms and gymnosperms, respectively.

	Leaf Length		Leaf Width		LMA		LT		LD_DM/LV_		CD		VBD		SL		Biomass		RMF		SMF		LMF		Root:shoot	
	C	Fert	C	Fert	C	Fert	C	Fert	C	Fert	C	Fert	C	Fert	C	Fert	C	Fert	C	Fert	C	Fert	C	Fert	C	Fert
**Leaf length**	1	1																								
**Leaf width**	0.055	−0.066	1	1																						
**LMA**	−0.294	−0.426	0.432	0.451	1	1																				
**LT**	0.308	0.427	−0.894 **	−0.907 **	−0.720 **	−0.708 **	1	1																		
**LD_DM/LV_**	−0.217	−0.341	0.883 **	0.890 **	0.792 **	0.799 **	−0.986 **	−0.981*	1	1																
**CD**	0.547	0.420	0.856 **	0.863 **	0.305	0.302	−0.614 *	−0.632 *	0.665 *	0.678 *	1	1														
**VBD**	−0.202	−0.289	0.816 **	0.846 **	0.867 **	0.850 **	−0.958 **	−0.949 **	0.986 **	0.988 **	0.630 *	0.680 *	1	1												
**SL**	−0.055	−0.188	0.953 **	0.967 **	0.683 *	0.662 *	−0.960 **	−0.965 **	0.978 **	0.974 **	0.793 **	0.804 **	0.945 **	0.951 **	1	1										
**Biomass**	−0.757 **	−0.770 **	0.520	0.549	0.633 *	0.319	−0.787 **	−0.715 **	0.740 **	0.596 *	0.080	0.105	0.706 *	0.502	0.632 *	0.564	1	1								
**RMF**	−0.686 *	−0.637 *	0.150	−0.246	308	0.500	−0.386	−0.085	339	0.127	−0.230	−0.506	0.295	0.119	0.240	−0.065	0.562	0.159	1	1						
**SMF**	0.791 **	0.926 **	−0.324	−0.259	−0.721 **	−0.579 *	0.672 *	0.592 *	−0.651 *	−0.535	0.93	0.217	−0.653 *	−0.480	−0.506	−0.393	−0.868 **	−0.780 **	−0.753 **	−0.688 *	1	1				
**LMF**	−0.591 *	−0.708 *	0.350	0.576	0.800 **	0.352	−0.664 *	−0.739 **	0.672 *	0.623 *	0.053	0.152	0.712 **	0.554	0.543	0.598*	0.807 **	0.932 **	0.279	0.057	−0.841 **	−0.764 **	1	1		
**Root: Shoot**	−0.677 *	−0.642 *	0.128	−0.207	0.328	0.537	−0.374	−0.126	0.332	0.170	−0.238	−0.470	0.294	0.163	0.229	−0.023	0.546	0.179	0.998 **	0.997 **	−0.752 **	−0.700 *	0.277	0.076	1	1

Note: *, ** indicates significant differences between treatments at 0.05 and 0.01 levels (in bold), respectively; changes of significance of correlations between N deposition levels are highlighted by grey shading.

## Supplementary Materials

The following are available online at https://www.mdpi.com/2223-7747/9/9/1231/s1, Table S1: plant biomass and its distribution to organs, Table S2: the ANOVA results of tree species, N deposition level, and their interaction on biomass parameters, Figure S1: increase in plant height., Figure S2: increase in collar diameter, Table S3: Pearson’s correlation coefficients of angiosperms’ leaf morphological and anatomical traits and biomass growth/parameters, Table S4: Pearson’s correlation coefficients of gymnosperms’ leaf morphological and anatomical traits and biomass growth/parameters.
