# Supplementary material for: Effects of Increased N Deposition on Leaf Functional Traits of Four Contrasting Tree Species in Northeast China
Source: Plants (Basel). 2020 Sep 18;9(9):1231. doi: 10.3390/plants9091231 (PMC7570078; doi:10.3390/plants9091231)
Supplement: Supplementary file 1 [file plants-09-01231-s001.pdf]

plants

Effects of Increased N Deposition on Leaf Functional Traits of Four Contrasting Tree Species in Northeast China

Attaullah Khan [1,†], Sun Jingjue [1,†], Nowsherwan Zarif [1,2], Kashif Khan [1], Muhammad Atif Jamil [1], Yang Lixue [1,*], Brent Clothier [3], and Boris Rewald [4]

[1] Key Laboratory of Sustainable Forest Ecosystem Management-Ministry of Education, School of Forestry, Northeast Forestry University, Harbin 150040, Heilongjiang, P. R. China; khan.aup252@gmail.com (A.K); jue_mz@126.com (S.J); noshu2002@gmail.com (N.Z); kashifkhanses@gmail.com (K.K); ati_eagle2007@live.com (M.J); ylx_0813@163.com (Y.L)
[2] Pakistan Forest Institute Peshawar, Pakistan (PFI)
[3] Sustainable Production, New Zealand Institute for Plant & Food Research Limited, Tennent Drive, Palmerston North, 4474, New Zealand; Brent.Clothier@plantandfood.co.nz (B.C)
[4] Forest Ecology, Dept. Forest and Soil Sciences, University of Natural Resources and Life Sciences Vienna, Peter-Jordan-Straße 82, 1190 Vienna, Austria; boris.rewald@boku.ac.at (B.R)
*Corresponding Author: (email: ylx_0813@163.com)
† A.K. and S.J. contributed equally to this work.

Supplementary Materials

Supplementary Table S1 The ANOVA results of tree species, N deposition level, and their interaction on biomass parameters of two-year-old seedlings of *Fraxinus mandshurica*, *Tilia amurensis* (both Angiosperms), *Pinus koraiensis* and *Larix gmelinii* (both Gymnosperms) in NE China at two treatments. Treatments are control (C; no additional nitrogen (N) deposition) and 10 g N m^{-2} yr^{-1} (Fert) additional N deposition. RMF; root mass fraction, SMF, stem mass fraction, LMF; leaf mass fraction, df degrees of freedom

Source of variation	df	Plant biomass	RMF	SMF	LMF	Root: shoot
Species (Spec)	3	**<0.001**	**<0.001**	**<0.001**	**<0.001**	**<0.001**
N deposition	1	**0.001**	**0.001**	0.387	**0.001**	**0.001**
Spec × N	3	0.289	0.160	0.045	0.048	0.270

P-values in the bold indicates significant effects

Supplementary Table S2 Plant biomass and its distribution to organs of two-year-old seedlings of *Fraxinus mandshurica, Tilia amurensis, Pinus koraiensis*, and *Larix gmelinii* at control (C; no additional nitrogen (N) deposition) and after 10 g N m^{-2} yr^{-1} (Fert) additional N deposition in NE China. See Supplementary Table 1 for 2-way ANOVA results; significant differences between N deposition levels per species are indicated by different lower-case letters (Tukey's HSD post hoc; $P < 0.05$; mean±SE); root mass fraction (RMF), stem mass fraction (SMF), leaf mass fraction (LMF)

**Species**	**N deposition level**	**Plant biomass (g)**	**RMF**	**SMF**	**LMF**	**Root: shoot**
F. mandshurica	C	32.1±2.26 a	0.44±0.03 a	0.22±0.01 a	0.34±0.03 a	0.80±0.08 a
	Fert	40.3±2.86 b	0.40±0.02 b	0.23±0.00 a	0.37±0.02 b	0.67±0.05 b
T. amurensis	C	25.9±1.04 a	0.41±0.01 a	0.31±0.01 a	0.28±0.01 a	0.70±0.03 a
	Fert	40.0±2.81 b	0.31±0.02 b	0.32±0.00 a	0.38±0.02 b	0.45±0.05 b
P. koraiensis	C	11.7±2.65 a	0.37±0.01 a	0.39±0.01 a	0.24±0.01 a	0.58±0.01 a
	Fert	19.9±2.92 b	0.32±0.01 b	0.41±0.00 a	0.27±0.00 b	0.48±0.01 b
L. gmelinii	C	21.7±1.82 a	0.43±0.01 a	0.31±0.01 a	0.26±0.01 a	0.77±0.03 a
	Fert	37.2±2.02 b	0.36±0.01 b	0.29±0.00 a	0.35±0.01 b	0.57±0.02 b

Note: The different letters in the same line indicate significant differences among the N deposition levels ($P<0.05$)

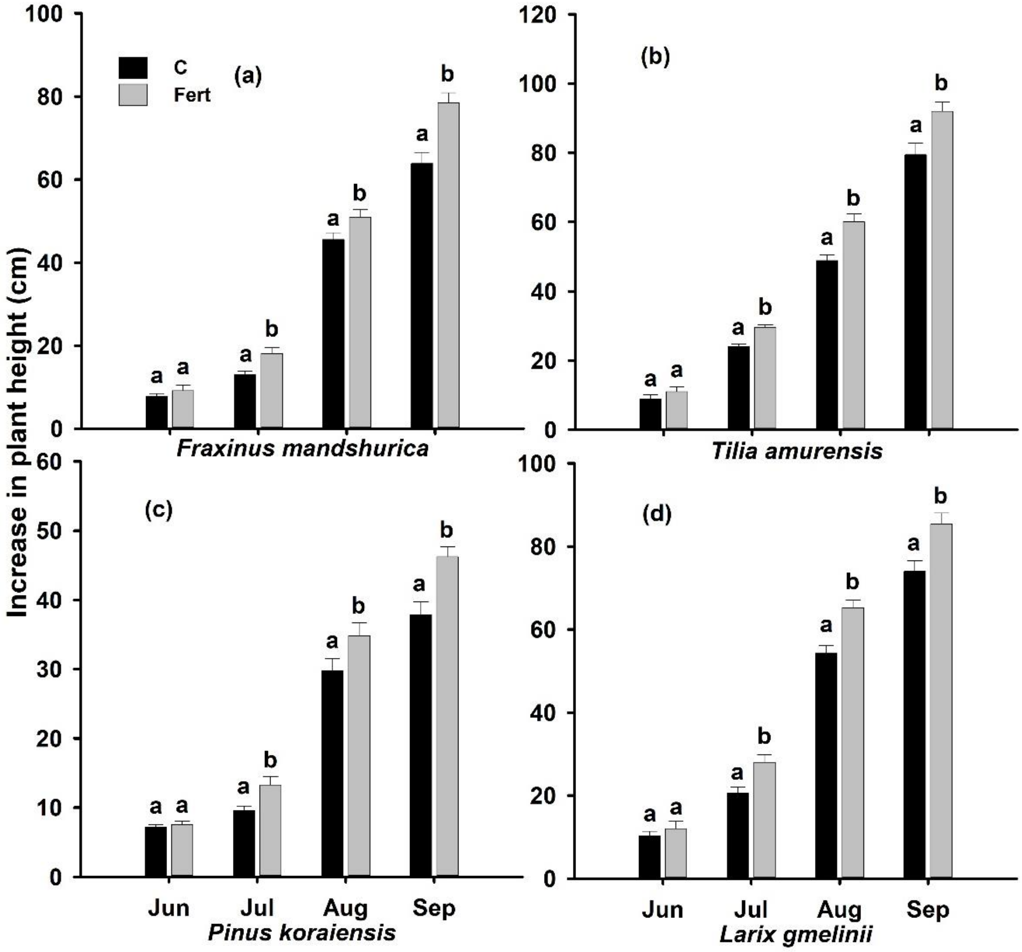

Supplementary Figure S1 Increase in plant height (cm; Jun-Sept. 2018) in *Fraxinus mandshurica* (a), *Tilia amurensis* (b), *Pinus koraiensis* (c), and *Larix gmelinii* (d) at control (C; no additional nitrogen (N) supply; black bars) and after 10 g N m^{-2} yr^{-1} (Fert; grey bars) additional N deposition in NE China. Within species, significant differences between N deposition levels are indicated by different lower-case letters (Tukey's HSD post hoc; $P < 0.05$; mean±SE)

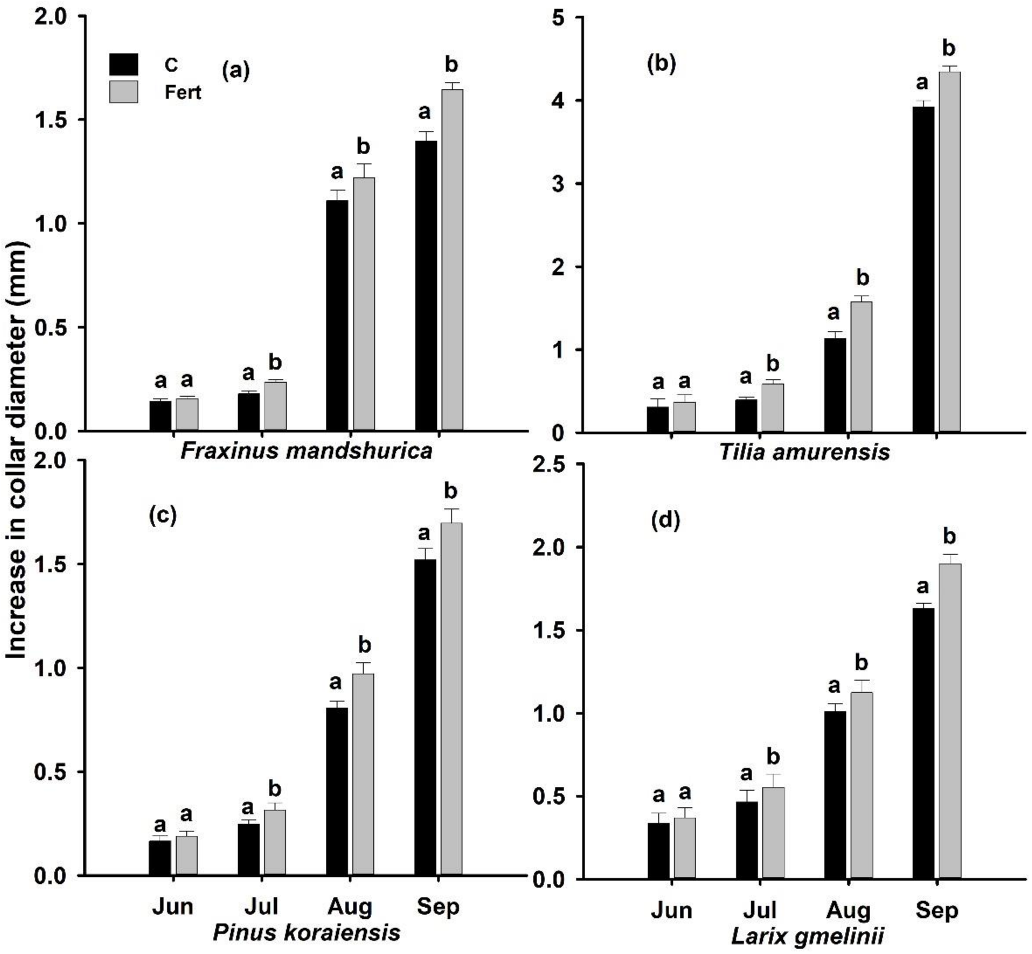

Supplementary Figure S2 Increase in collar diameter (cm; Jun-Sept. 2018) in *Fraxinus mandshurica* (a), *Tilia amurensis* (b), *Pinus koraiensis* (c), and *Larix gmelinii* (d) at control (C; no additional nitrogen (N) supply; black bars) and after 10 g N m^{-2} yr^{-1} (Fert; grey bars) additional N deposition in NE China. Within species, significant differences between N deposition levels are indicated by different lower-case letters (Tukey's HSD post hoc; $P < 0.05$; mean±SE)

Supplementary Table S3 Pearson's correlation coefficients of leaf morphological and anatomical traits and biomass growth /parameters of two-year-old saplings of angiosperms (*Fraxinus mandshurica, Tilia amurensis*) at control (C; no additional deposition) and after 10 g N m^{-2} yr^{-1} (Fert) additional nitrogen deposition in NE China. Abbreviations: leaf mass per area (LMA), leaf thickness (LT), leaf density measured ($LD_{DM/LV}$), conduit diameter (CD), vascular bundle diameter (VBD), palisade mesophyll thickness (PMT), spongy mesophyll thickness (SMT), adaxial epidermis (ADE), abaxial epidermis (ABE), stomata pore length (SL), root mass fraction (RMF), stem mass fraction (SMF), and leaf mass fraction (LMF)

	Leaf length		Leaf width		LMA		LT		$LD_{LMA/LT}$		CD		VBD		PMT		SMT		ADE		ABE		SL		Biomass		RMF		SMF		LMF		Root: shoot	
	C	Fert	C	Fert	C	Fert	C	Fert	C	Fert	C	Fert	C	Fert	C	Fert	C	Fert	C	Fert	C	Fert	C	Fert	C	Fert	C	Fert	C	Fert	C	Fert	C	Fert
Leaf length	1	1																																
Leaf width	.973**	.983**	1	1																														
LMA	-.788	-.802	-.904*	-.898*	1	1																												
LT	-.884*	-.873*	-.965**	-.948**	.984**	.991**	1	1																										
$LD_{LMA/LT}$	.798	.191	.675	.024	-.322	.379	-.486	.253	1	1																								
CD	.998**	.991**	.982**	.985**	-.814*	-.827*	-.901*	-.889*	.762	.109	1	1																						
VBD	-.483	-.263	-.648	-.429	.897*	.764	.808	.673	.117	.866*	-.526	-.322	1	1																				
PMT	-.929**	-.916*	-.987**	-.974**	.959**	.974**	.994**	.995**	-.570	.171	-.941**	-.927**	.749	.605	1	1																		
SMT	-.735	-.718	-.862*	-.831*	.993**	.984**	.961**	.956**	-.229	.517	-.765	-.750	.935**	.846*	.928**	.929**	1	1																
ADE	-.487	-.477	-.662	-.628	.917*	.901*	.834*	.839*	.051	.688	-.522	-.516	.969**	.954**	.772	.784	.947**	.938**	1	1														
ABD	.410	.546	.191	.384	.221	.059	.051	-.069	.747	.805	.364	.506	0.520	.610	-.053	-.165	.284	.166	.548	.464	1	1												
SL	.977**	.993**	.915*	.964**	-.658	-.757	-.783	-.836*	.906*	.267	.964**	.985**	-.292	-.186	-.844*	-.884*	-.589	-.657	-.315	-.413	.552	.594	1	1										
Biomass	-.732	-.133	-.749	-.034	.751	-.194	.781	-.108	-.446	-.679	-.724	-.004	.603	-.508	.786	-.063	.755	-.252	.664	-.351	-.170	-.435	-.660	-.127	1	1								
RMF	-.392	-.698	-.491	-.795	.494	.908*	.485	.890*	-.206	.393	-.435	-.757	.375	.733	.475	.869*	.477	.870*	.395	.844*	.284	.102	-.362	-.688	.004	-.474	1	1						
SMF	.883*	.960**	.957**	.992**	-.956**	-.925**	-.984**	-.964**	.549	-.059	.895*	.971**	-.739	-.510	-.982**	-.982**	-.929**	-.865*	-.801	-.693	-.019	.310	.805	.939**	-.808	.047	-.517	-.853*	1	1				
LMF	-.721	-.182	-.731	-.045	.728	-.276	.766	-.181	-.477	-.659	-.701	-.085	.567	-.598	.772	-.112	.710	-.299	.624	-.520	-.193	-.684	-.653	-.168	.928**	.833*	-.162	-.566	-.761	.052	1	1		
Root:shoot	-.409	-.693	-.513	-.793	.520	.911*	.511	.893*	-.213	.393	-.452	-.752	.399	.727	.500	.871*	.502	.872*	.420	.844*	.294	.116	-.376	-.684	.020	-.474	.999**	.997**	-.540	-.846*	-.135	-.573	1	1

Note: *, ** indicates significant differences between N deposition levels at 0.05 and 0.01 levels (in bold), respectively; changes of significance of correlation between N deposition levels are highlighted by frames

Supplementary Table S4 Pearson's correlation coefficients of leaf morphological and anatomical traits and biomass growth /parameters of two-year-old saplings of gymnosperms (*P. koraiensis*, *L. gmelinii*) at control (C; no additional deposition) and after 10 g N m^{-2} yr^{-1} (Fert) additional nitrogen deposition in NE China. Abbreviations: leaf mass per area (LMA), leaf thickness (LT), leaf density estimated ($LD_{DM/LV}$), conduit diameter (CD), vascular bundle diameter (VBD), mesophyll thickness (MT), resin duct diameter (RD), stomata pore length (SL), root mass fraction (RMF), stem mass fraction (SMF), and leaf mass fraction (LMF)

	Leaf length		Leaf width		LMA		LT		$LD_{DM/LV}$		CD		VBD		MT		RD		SL		Biomass		RMF		SMF		LMF		Root:shoot	
	C	Fert	C	Fert	C	Fert	C	Fert	C	Fert	C	Fert	C	Fert	C	Fert	C	Fert	C	Fert	C	Fert	C	Fert	C	Fert	C	Fert	C	Fert
**Leaf length**	1	1																												
**Leaf width**	.973**	.964**	1	1																										
**LMA**	.979**	.984**	.990**	.983**	1	1																								
**LT**	.991**	.989**	.982**	.957**	.995**	.982**	1	1																						
**$LD_{DM/LV}$**	.895*	.916*	.967**	.962**	.946**	.958**	.921**	.887*	1	1																				
**CD**	.994**	.988**	.960**	.933**	.979**	.969**	.990**	.996**	.870*	.863*	1	1																		
**VBD**	.983**	.992**	.995**	.976**	.995**	.990**	.990**	.994**	.941**	.918**	.979**	.986**	1	1																
**MT**	-.103	-.279	.092	-.120	-.021	-.203	-.082	-.367	.293	.082	-.185	-.410	.001	-.306	1	1														
**RD**	.984**	.958**	.946**	.899*	.963**	.935**	.976**	.981**	.837*	.796	.994**	.986**	.971**	.969**	-.218	-.529	1	1												
**SL**	.955**	.987**	.990**	.977**	.986**	.992**	.975**	.968**	.949**	.963**	.949**	.960**	.990**	.985**	.065	-.165	.917*	.910*	1	1										
**Biomass**	-.958**	-.991**	-.967**	-.966**	-.955**	-.984**	-.963**	-.989**	-.919**	-.909*	-.932**	-.984**	-.959**	-.998**	-.072	.324	-.896*	-.963**	-.965**	-.985**	1	1								
**RMF**	-.833*	-.802	-.751	-.775	-.815*	-.832*	-.844*	-.878*	-.590	-.677	-.878*	-.873*	-0.806	-.843*	.547	.573	-.922**	-.902*	-.791	-.769	.763	.829*	1	1						
**SMF**	.844*	.910*	.739	.824*	.807	.887*	.837*	.950**	.576	.718	.893*	.964**	0.799	.918**	-.599	-.615	.937**	.987**	.749	.858*	-.709	-.917**	-.966**	-.940**	1	1				
**LMF**	-.594	-.923**	-.466	-.810	-.520	-.872*	-.544	-.942**	-.352	-.705	-.636	-.964**	-0.518	-.913*	.533	.607	-.668	-.984**	-.399	-.863*	.341	.920**	.563	.859*	-.758	-.982**	1	1		
**Root:shoot**	-.826*	-.798	-.747	-.772	-.812*	-.831*	-.838*	-.875*	-.587	-.677	-.873*	-.870*	-0.803	-.840*	.545	.570	-.918**	-.899*	-.790	-.768	.755	.826*	1.000**	1.000**	-.963**	-.937**	.555	.855*	1	1

Note: *, ** indicates significant differences between N deposition levels at 0.05 and 0.01 levels (in bold), respectively; changes of significance of correlations between N deposition levels are highlighted by frames

plants

© 2020 by the authors. Submitted for possible open access publication under the terms and conditions of the Creative Commons Attribution (CC BY) license (http://creativecommons.org/licenses/by/4.0/).
